# Transcriptional repression shapes the identity and function of tissue macrophages

**DOI:** 10.1002/2211-5463.13269

**Published:** 2021-08-14

**Authors:** Krisztian Bene, Laszlo Halasz, Laszlo Nagy

**Affiliations:** ^1^ Department of Biochemistry and Molecular Biology Nuclear Receptor Research Laboratory Faculty of Medicine University of Debrecen Debrecen Hungary; ^2^ Departments of Medicine and Biological Chemistry Johns Hopkins University School of Medicine Institute for Fundamental Biomedical Research Johns Hopkins All Children's Hospital St. Petersburg FL USA

**Keywords:** epigenome, genome, macrophage, repression, tissue‐resident, transcription

## Abstract

The changing extra‐ and intracellular microenvironment calls for rapid cell fate decisions that are precisely and primarily regulated at the transcriptional level. The cellular components of the immune system are excellent examples of how cells respond and adapt to different environmental stimuli. Innate immune cells such as macrophages are able to modulate their transcriptional programs and epigenetic regulatory networks through activation and repression of particular genes, allowing them to quickly respond to a rapidly changing environment. Tissue macrophages are essential components of different immune‐ and nonimmune cell‐mediated physiological mechanisms in mammals and are widely used models for investigating transcriptional regulatory mechanisms. Therefore, it is critical to unravel the distinct sets of transcription activators, repressors, and coregulators that play roles in determining tissue macrophage identity and functions during homeostasis, as well as in diseases affecting large human populations, such as metabolic syndromes, immune‐deficiencies, and tumor development. In this review, we will focus on transcriptional repressors that play roles in tissue macrophage development and function under physiological conditions.

## Graphical abstract

Tissue macrophages are essential cellular components in maintaining tissue homeostasis and integrity through their effector functions. Tissue‐resident macrophages such as the alveolar, splenic red pulp, intestinal macrophages, and microglia have overlapping function. The identity and function of tissue‐resident macrophage subtypes are orchestrated by distinct sets of transcriptional activator and repressor proteins. In this review, we focus on repressors.

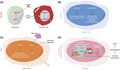

**Abbreviations**
(G)M‐CSF: (Granulocyte‐) macrophage colony‐stimulating factor
BACH1/2: BTB and CNC homology 1/2
BCL‐6: B‐cell lymphoma 6 protein
ChIP: chromatin immunoprecipitation
eRNA: enhancer RNA
GPS2: G protein pathway suppressor 2
HDAC: histone deacetylase
HMT: histone methyl transferase
IFN: interferon
IRF4/8: interferon regulatory factor 4/8
LDTF: lineage-determining transcription factor
LXR: liver X receptor
NCoR: nuclear receptor corepressor
NfκB: nuclear factor kappa-light-chain-enhancer of activated b cells
NRF2: nuclear factor erythroid 2-related factor 2
PPARγ: peroxisome proliferator-activated receptor gamma
PRR: pattern recognition receptor
RXR: retinoid X receptor
SDTF: signal-dependent transcription factor
SMRT: silencing mediator for retinoid and thyroid hormone receptors
STAT: signal transducer and activator of transcription

Macrophages belong to the innate immune system and represent a highly plastic immune cell population at both transcriptional and functional level [1, 2, 3]. Macrophages possess several effector and regulatory functions in immunity including phagocytosis, inflammation, cell killing, antigen presentation, immune complex delivery, and tissue regeneration. These functions are determined by the local tissue and organ demands under physiological conditions. Resident macrophages as accessory cells are able to support the activity of local parenchymal cells to maintain the integrity and the physiological function of the local tissue and organ [2].

The effector functions of macrophages are tightly regulated by environmental cues such as infectious agents, cytokines, chemokine, growth factors, lipids, and metabolites, enabling macrophages to rapidly adapt and respond to a given microenvironment and multiple stimuli [4]. A major goal of macrophage biology research is to uncover the molecular mechanisms of macrophage development and polarization and link signaling pathways to specific physiological and pathological processes [5].

Notably, an emerging number of studies demonstrate the potential role of distinct transcription factors in tissue‐resident macrophage development and function. These cellular processes involve lineage‐determining transcription factors (LDTF), signal‐dependent TFs (SDTF), and transcriptional repressor proteins. Nuclear receptors such as peroxisome proliferator‐activated receptor gamma (PPARγ) and LXRα regulate macrophage functions in a lipid‐rich environment. Surprisingly, the systematic analyses of tissue‐resident macrophage enhancer landscapes also detected the DNA‐binding motifs of these transcription factors [3, 6, 7], suggesting that these lipid‐sensing nuclear receptors may also act as LDTFs in different tissue‐resident macrophages and may also act in a ligand‐independent manner [8].

Although macrophages are highly plastic and are able to adapt to different polarizing environments, their differentiation capacity and transcription are restricted. An increasing amount of data support the role of tissue environment in cell fate determination by establishing tissue‐resident macrophage enhancer activity, gene expression, and phenotype [3]. Thus, the phenotypes of tissue‐resident macrophages are most likely a combination of differentiation and polarization programs including tissue‐specific repressive mechanisms.

## Tissue macrophages

Macrophage function consists of increased levels of endocytosis and lysosomal biogenesis, as well as expression of a wide range of pattern recognition receptors (PRRs) and polymorphic antigen‐presenting molecules. These processes play an essential role in the first line of defense against pathogens and in ensuring tissue integrity [9].

Tissue macrophages share a wide range of functions in the central and peripheral lymphoid tissues but these cells also exhibit large differences in their enhancer landscape [3]. This suggests that functions can be tightly regulated by transcriptional regulatory mechanisms determined by the ontogeny of origin and by the tissue microenvironment as well.

Anatomically distinct areas of the mammalian body are usually associated with different physiological processes; thus, different organs require distinct functional properties of resident macrophages as accessory cells of the local parenchymal cells [2]. For example, endocytosis is one of the most important functions of tissue macrophages. Subcapsular sinus and germinal center macrophages are specialized for the uptake of immunocomplexes and for the phagocytosis of large amounts of apoptotic B cells in the germinal center, respectively [2, 9]. The phagocytic system of the splenic red pulp and bone marrow macrophages are responsible for clearing injured and aging red blood cells and also for neutralizing free labile heme and contribute to the recirculation of iron [10, 11]. As a first line of defense, the mucosa‐associated macrophages including alveolar, gut, and peritoneal macrophages can act against the microbiota and their products and protect against invading pathogenic microbes [12, 13, 14, 15]. In addition, alveolar macrophages are responsible for the proteolysis and the removal of surfactant in the lungs. Osteoclasts are highly specialized in bone remodeling and maintaining the hematopoietic stem cell niche [16], while microglia are a unique resident macrophage subtype, having a major role in the development and function of the central nervous system (CNS) [17].

Some tissue macrophage populations with self‐renewal ability [18] are derived from the yolk sac. This set of macrophages include microglia [17] and alveolar macrophages [19] and abundant gut‐resident macrophage subtypes are maintained independently of monocytes [20]. In contrast, a group of macrophages is derived from both fetal and adult hematopoiesis including peritoneal macrophages, red pulp macrophages of the spleen, and Kupffer cells of the liver. This indicates that hematopoiesis in adulthood plays a limited role in maintaining tissue macrophage subpopulations. The plasticity of tissue macrophages is further supported by recent *in vivo* models. For example, peritoneal macrophages can re‐populate the liver rapidly and show an alternative macrophage program in a sterile liver injury model [21]. The circulating monocytes are also able to occupy most of the tissues and acquire their phenotype from the local tissue microenvironment. Notably, the transcription program of monocyte‐derived macrophages is limited as compared to that of tissue‐resident macrophages [18].

## Determining the macrophage lineage and polarization signals

The ontogenic origin of macrophages, namely monocyte‐derived and embryonic tissue‐resident macrophages also determines differentiation and polarization programs in macrophages [5, 22]. The lineage‐determining transcription factors (LDTF) including PU.1 [23], MYB, c‐MAF, MAFB [24, 25], CEBP [26], IRF8 [27], and AP‐1 determine the first layer for macrophage identity and establish a core, irreversible macrophage program and is shared by all subsets of macrophages. The PU.1 transcription factor binds to and engages macrophage‐specific enhancers enabling them to provide access to other transcriptional regulators [28]. Expression of PU.1 and the universal macrophage‐specific gene expression module is maintained by the CSF‐1 receptor and its ligands [23], including M‐CSF and interleukin (IL)‐34. In addition, the development of macrophages is driven, at least partly, by local niche signals and transcription factors and is associated with a tissue‐specific enhancer landscape regardless of the source of cell origin. Local niche signals include macrophage‐polarizing factors such as cytokines (interleukin/IL‐4, interferon/IFN γ), metabolites (RA), and growth factors (TGF‐ß [29], M‐CSF) as well as microbe‐derived factors (lipopolysaccharide, butyrate, indoles) [5]. These extrinsic macrophage‐polarizing signals involve type 1 macrophage (M1)‐associated IFNγ and Toll‐like receptor (TLR) ligands while M2 cells are associated with IL‐4 and IL‐13 cytokines [5]. The reversible, macrophage‐polarizing stimuli are widely used in macrophage biology *in vitro*; nevertheless, we have only a moderate size of data from *in vivo* model systems studying macrophage differentiation and polarization at genomic and epigenomic levels. Therefore, it is crucial to characterize the interplay of LDTFs and SDTFs in distinct tissue macrophage‐polarizing microenvironments and to uncover indispensable transcriptional activator and repressor mechanisms in macrophage development, polarization, and function.

## Transcriptional repressor mechanisms

The regulation of gene expression is controlled at the genomic level by proximal [30] and distal [31, 32, 33] regulatory elements as well as by epigenomic mechanisms [7] and by chromatin remodeling [31, 34]. Enhancers and promoters are fundamental determinants of gene expression, and in contrast to promoter sequences, enhancers are greater in number and can regulate gene expression at multiple levels [35]. Tissue macrophages have unique enhancer landscape [3, 28]; however, complex macrophage lineage‐determining environmental factors and polarizing signals can modify the transcriptional program of developing myeloid cells and macrophages. Enhancer sequences enable distinct regulatory transcriptional mechanisms by recruiting cofactors and by chromatin remodeling. Importantly, most of the SDTFs are more enriched at enhancers than promoter sequences, indicating that enhancer sequences are essential in the diverse and dynamic regulation of gene transcription [35].

The precise regulation at enhancer regions is based on the significant enrichment of DNA motif recognition by different transcription factors and repressors associated with or without coregulators at the identical motif sequences [36]. The protein family of coregulators consists of corepressors and coactivators in a context‐specific manner [1, 37]. The direct repression mechanism establishes transcriptional machinery involving transcription factors and corepressor complexes containing histone deacetylase (HDAC) and/or methyl transferases targeting histone‐bound gene promoter and enhancer sequences. Alternatively, transrepression orchestrates a nuclear transcription factor complex inhibiting the activity of another transcription factor such as IRF3, JUN, nuclear factor kappa‐light‐chain‐enhancer of activated b cells (NFκB)‐p65, SMAD3/4, signal transducer and activator of transcription (STAT)5/6, and T‐bet by protein–protein interactions [38]. For example, the mechanism of transrepression is a feature of nuclear hormone receptors including glucocorticoid receptors and PPARγ [39, 40]. Stimulating signals can initiate the exchange of corepressor and coactivator complexes at genomic regulatory elements to establish the transcriptional machinery. The mechanism of action of transcription factors with coregulators and chromatin is gene‐ and cell type‐specific [33] and, more importantly, enables a multilevel adaptation to the extracellular environment such as the alternative activation program of macrophages [41].

The regulation of gene expression can be mediated by epigenomic mechanisms including histone modifications such as histone methylation and acetylation. Promoters are marked by H3K4m3, while enhancers exhibit high levels of H3K4m1 and H3K4m2 [1, 37]. Active and repressed regulatory elements of the DNA are associated with H3K27Ac and H3K27m3, respectively. The available enhancer repertoire has a fundamental role in determining tissue‐resident macrophage identity by binding with certain lineage‐determining factors [3, 33], resulting in chromatin opening, enhancer activation, and new loop formation between promoters and enhancers [3].

The active transcriptional repression by recruiting HDAC and histone methyl transferase (HMT) enzymes involves transcription factors such as BACH1 [42] and B‐cell lymphoma 6 protein (Bcl‐6) [43] which can occupy specific regulatory elements of the genome and can inhibit the transcription of target genes including macrophage *Hmox1* [42, 44, 45] and *Il6* [43], respectively. Intriguingly, known transcriptional activators such as STAT proteins can also act as transcriptional repressors. In the case of active transcriptional repression, we have previously shown that the IL‐4 activated transcription factor STAT6 reduces the binding of transcriptional coactivator and RNA‐polymerase II as well as by H3K27 acetylation at enhancer regions regulating the genes of inflammatory responsiveness in murine macrophages [41]. In human macrophages, it was reported that the inflammatory IFNγ cytokine inhibits expression of IL‐4 target genes by enhancer of zeste homolog 2 (Ezh2)‐mediated H3K27 trimethylation at a subset of IL‐4 target gene promoters including the gene coding the anti‐inflammatory transcription factor PPARγ [46].

Active transcriptional repression can be induced and maintained by recruiting nuclear receptor corepressor (NCoR)/silencing mediator for retinoid and thyroid hormone receptors (SMRT) corepressor complexes involving HDAC3 and CoREST containing both histone demethylase and deacetylase enzymes such as HDAC1,2 [47, 48]. NCoR/SMRT and CoREST corepressor complexes can mediate transcriptional repression in macrophages and play essential role in inflammation, macrophage polarization, and lipid metabolism [37]. Recent studies indicated that NCoR/SMRT complexes are required for basal repression of a subset of NF‐κB and AP‐1 target genes, with loss of NCoR resulting in a partially activated phenotype in macrophages. A set of genes encoding inflammatory cytokines and chemokines is de‐repressed in NCoR‐deficient macrophages. Alternatively, these genes are also regulated by transrepression by PPARγ, suggesting a possible role for NCoR in this process and other transcription factors such as NFκB, AP‐1, and STAT1, which proteins are also involved [49, 50]. Further studies confirmed the role of NCoR complexes in pathological conditions where SMRT and NCoR complexes can also prevent autoimmune chronic inflammatory processes [43] and macrophage‐dependent metabolic disease [51].

Tissue macrophages are exposed to various stimuli in both homeostatic and pathophysiological conditions which require complex cell signaling and transcriptional mechanisms to rapidly adapt to the changing environment. These processes also include Rev‐Erb proteins such as Rev‐Erb‐α and Rev‐Erb‐ß, which play critical roles in orchestrating danger‐associated and macrophage‐polarizing signaling events. For example, Rev‐Erb proteins colocalize with master SDTFs such as p65, Fos, Smad3, and nuclear factor erythroid 2‐related factor 2 (Nrf2) at enhancers stimulated by a complex damage signals during wound healing [52]. Rev‐Erbs can recruit repressor complexes involving NCoR and HDAC3 proteins and establish a macrophage‐specific transcriptional repressor program [53], and Rev‐Erbs can inhibit the functions of distal enhancers that are targeted by macrophage LDTFs. Importantly, some enhancer RNA (eRNA) sequences play critical role in enhancer functions at specific genomic regions.

Macrophage functions are often linked to metabolic diseases such as obesity or type 2 diabetes. A potential connection between metabolic syndrome and the transcriptional repressor G protein pathway suppressor 2 (GPS2) was uncovered, where a defined set of inflammatory genes are repressed by GPS2 protein [54]. Moreover, the axis of the GPS2‐SMART corepressor complex together with *Ccl2* eRNA exacerbates inflammation by adipose tissue‐derived macrophages in obese mice [55]. This study further supports the functional role of eRNAs in connecting enhancer activity to inflammatory gene expression via modulating CBP‐mediated H3K27 acetylation and enhancer–promoter looping. However, further studies are required to solidify these initial reports and establish that eRNAs are functional components of gene expression regulation and if their activity is dependent on their sequence or not.

Our group recently summarized that nuclear hormone receptors could also act as transcriptional repressors in a ligand‐independent manner [56]. For example, NCoR and SMRT corepressors are the most common interacting partners of retinoid X receptor (RXR) in mediating transcriptional repression. This mechanism is important in the case of antitumor chemotherapies targeting RXR/RAR molecules; however, a large ratio of patients does not respond to these therapies. We have shown that deletion of RXR in myeloid cells enhances lung metastasis formation while not affecting primary tumor growth and that RXR deficiency leads to gene expression changes in the lung myeloid compartment. These changes show increased expression of prometastatic genes *Il1a*, *Il1b*, *Pdgfb*, *Sema4d*, *Igf1*, and *Ctss*, including key determinants of premetastatic niche formation. RXR‐deficient myeloid cells are also more efficient in promoting cancer cell migration and invasion. The repressive activity of RXR on such prometastatic genes is mediated primarily through direct DNA binding of the receptor along with NCoR and SMRT corepressors and is largely unresponsive to ligand activation [57].

The transcriptional program of tissue‐resident macrophage is adapted to the local environment and is regulated by unique transcriptional regulatory mechanisms

**Fig. 1:**
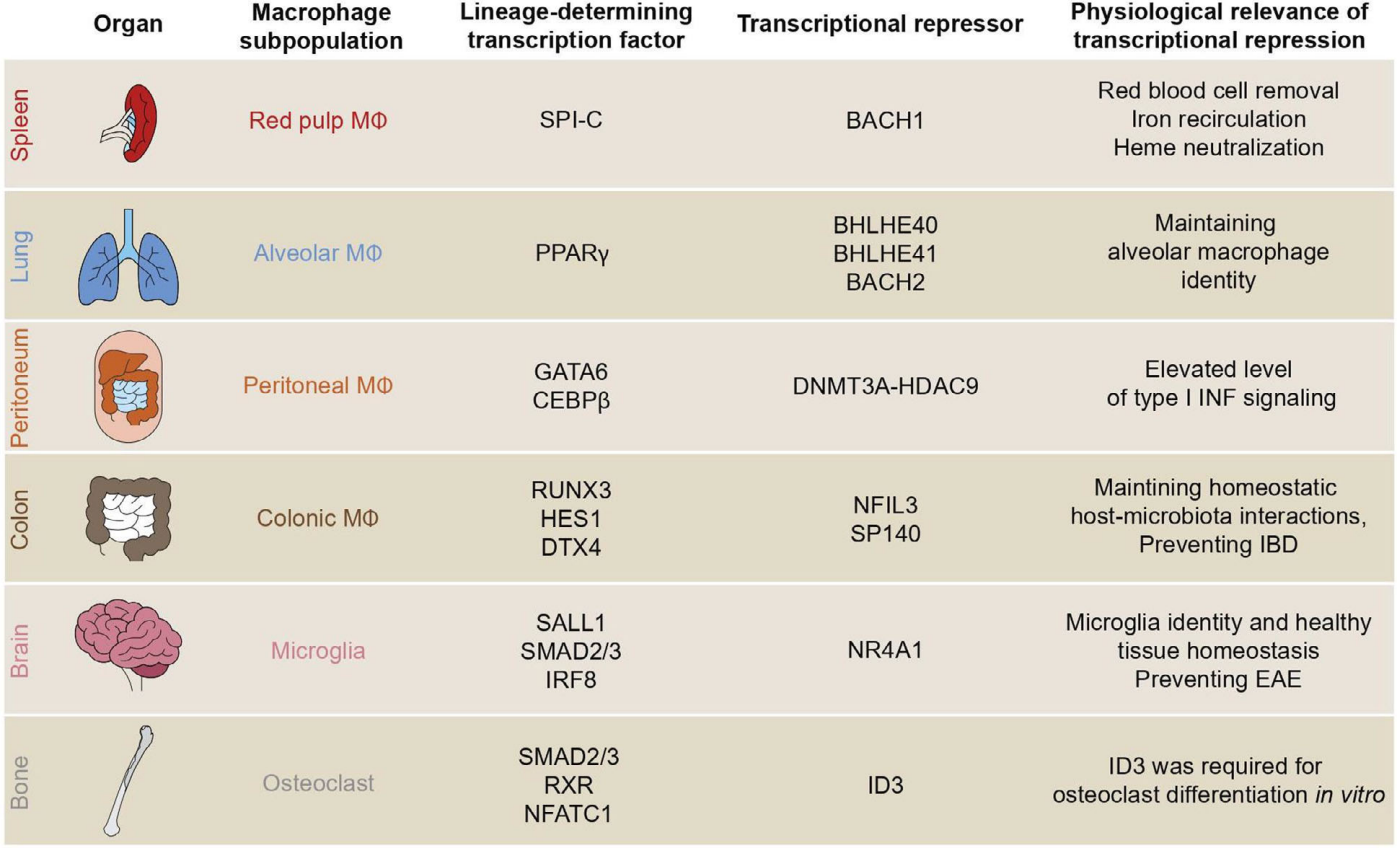
Transcription factors and transcriptional repressors of tissue macrophages. EAE, experimental autoimmune encephalomyelitis. IBD, inflammatory bowel syndrome.

Distinct tissue environment requires unique functional properties from accessory cells such as resident macrophages. Tissue‐resident macrophages support local, parenchymal ‘client’ cells and maintain homeostatic conditions and tissue integrity [2]. This mechanism involves different sets of genes regulated by resident macrophage LDTFs (Fig. 1). For example, the deletion of LDTFs induces deficiency in specific tissue‐resident macrophage subsets, such as Spi‐C in splenic red pulp macrophages [58], LXRα in splenic marginal zone macrophages [59], PPARγ in lung alveolar macrophages [19], GATA6 in peritoneal macrophages [60], and NR4A1 in microglia and thymus macrophages [61].

### Splenic red pulp macrophages

**Fig. 2:**
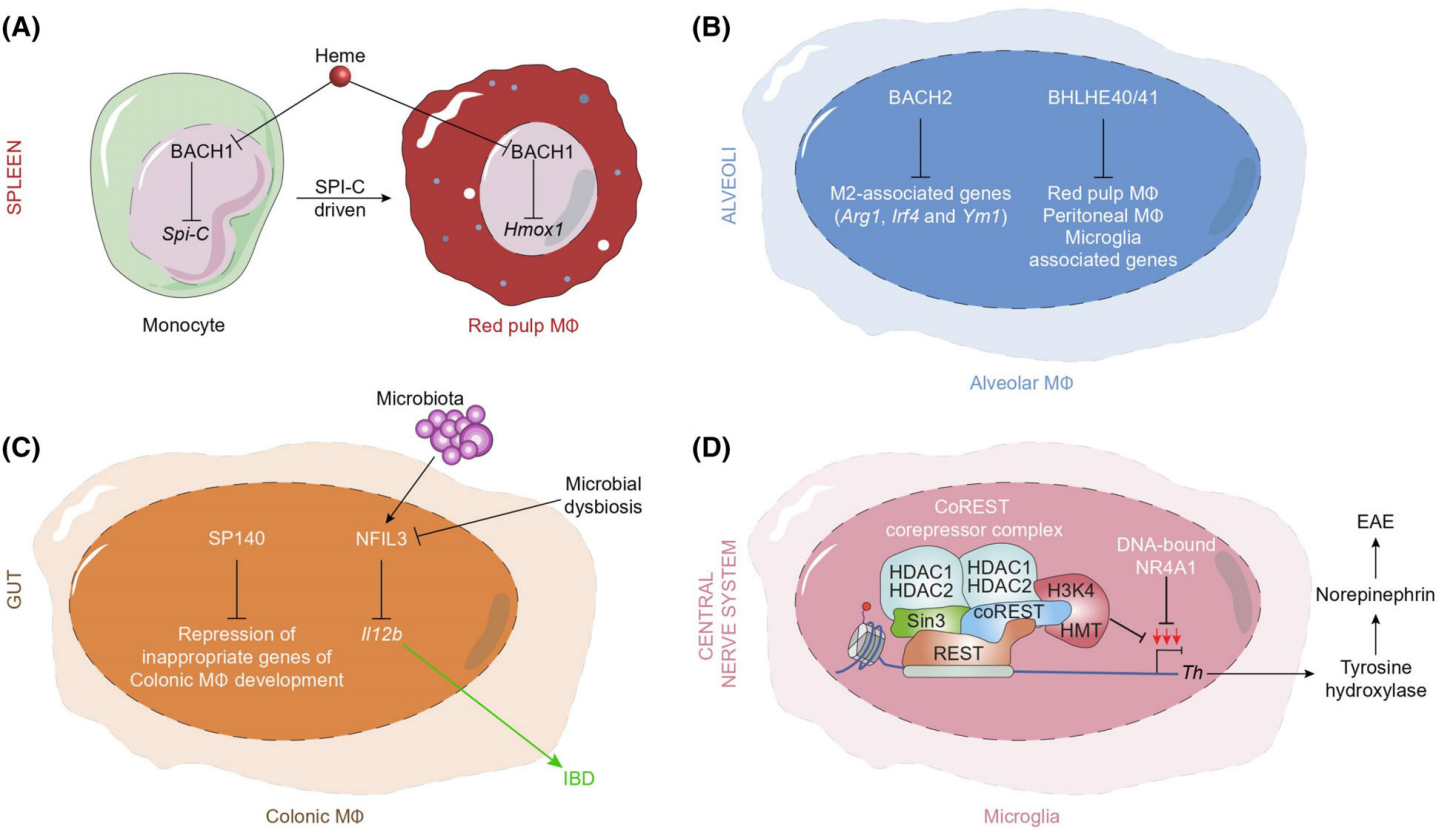
Molecular mechanisms of repressors pathways regulating tissue macrophage identity. (A) Free labile heme modifies the differentiation of monocytes in the spleen by the binding to the nuclear BACH1 protein. BACH1 protein directly represses genes such as Spi‐C, the master transcription factor of red pulp macrophages. In splenic red pulp macrophages, the de‐repression of other BACH1‐target gene Hmox1 coding heme‐oxygenase plays role in the neutralization of free labile heme in circulation. (B) In alveolar macrophages, BACH2 represses genes playing role in M2 macrophage functions and polarization. The BLHLE40/41 proteins repress lineage‐determining factors associated with peritoneal, red pulp macrophages and microglia. (C) SP140 protein represses genes involved in noncolonic macrophage development. NFIL3 supports the homeostatic host–microbiota interactions in the gut. (D) Microglia cell development and function is dependent on NR4A1, which limits the expression level of Th coding tyrosine hydroxylase by recruiting CoREST complex; thus, microglial NR4A1 prevents EAE.

The differentiation of macrophages populating the bone marrow and the spleen red pulp depends on the transcription factor Spi‐C as well the heme‐sensitive transcriptional repressor BACH1 [10, 58] (Fig. 2A). The Bach1 mRNA is highly expressed in subsets of monocytes, macrophages, neutrophils, and dendritic cells, whereas Bach2 mRNA is highly expressed in especially lymphocytes but also expressed in some subsets of myeloid cells [42]. BACH proteins belong to the CNC family of bZIP transcription factor superfamily together with nuclear factor erythroid (NRF)‐1,2,3 and P45 proteins. The bZIP superfamily also includes activator protein 1 (AP‐1), cAMP response element‐binding (CREB), C/EBP, MAF, and PAR protein families [42]. The bZIP transcription factors form dimeric interactions at DNA binding sites containing core sequences known as TPA response elements (TREs) or cAMP response elements (CREs). In differentiated red pulp macrophages, the presence of intracellular heme derepresses direct BACH1‐target genes including *Hmox1* coding anti‐inflammatory heme‐oxygenase (HO) 1 [11], *Spic* [10, 58], and *Slc40a1* coding ferroportin as well as glucose metabolism‐related genes including the pentose phosphate pathway [62]. The free heme is able to bind directly to the cysteine‐proline motifs‐enriched binding site of the chromatin‐bound BACH1, which leads to the translocation of BACH1 into the cytosol and induces the proteasomal degradation [63]. The repressor BACH1 and the transcriptional activator NRF2 competes for overlapping proximal and distal enhancer elements of genes that play roles in antioxidant and anti‐inflammatory responses leading a sensitive regulatory system to neutralize free heme [64, 65]. The increased expression level of HO1 in the cytosol enables the neutralization and the catabolism of the toxic heme into biliverdin [66], CO, and ferrous iron, which also have local physiological and immunostimulatory effects [67, 68]. Intriguingly, the homeostatic and pathologic concentration of free labile heme determines the outcome of myeloid differentiation in the bone marrow as well as the spleen red pulp in a SpiC‐, and BACH1‐dependent manner [10]. This study illustrates for the first time how metabolites, namely heme can mediate the differentiation of a tissue‐resident macrophage populations. Alternatively, the gene expression level of *Spic* can also be up‐regulated in a BACH1‐independent manner during inflammation resulting in attenuated macrophage response and increased iron efflux [10].

### Alveolar macrophages

After birth, alveolar macrophages (AMs) play an essential role in maintaining healthy lung homeostatic conditions. The development of AMs is mediated by alveolar satellite cell‐derived factors including granulocyte–macrophage colony‐stimulating factor (GM‐CSF). The growth factor GM‐CSF upregulates the nuclear receptor PPARγ [19], the nuclear repressor BACH2, and genes related to host defense (Fig. 2B). The activity of PPARγ in alveolar macrophages ensures the removal of the produced surfactants in the lungs and the repression of target inflammatory genes [69]. Notably, the GM‐CSF‐induced differentiation polarizes the alveolar macrophages into the classical, immunogenic M1 macrophage subtype [19]. The deficiency in GM‐CSF‐dependent PPARγ‐activity in AM leads to pulmonary alveolar proteinosis (PAP). Mice deficient for the BACH2 developed PAP‐like accumulation of surfactant proteins in the lungs, while AMs showed a normal expression of the genes involved in the GM‐CSF signaling. However, BACH2‐deficient AMs displayed an altered expression level of genes playing a role in chemotaxis, phagocytosis and lipid metabolism, and alternative macrophage (M2) activation program associated with the increased gene expression level of *Ym1* and *arginase‐1*, and the M2 regulator *Irf4* [69].

The helix‐loop‐helix transcriptional repressors BHLHE40 and BHLHE41 also determine alveolar macrophage (AM) identity and directly repress genes playing roles in determining AM signature [70]. In addition, AMs lacking these two transcription factors exhibited impaired proliferation. Notably, in the absence of competition with wild‐type cells, *Bhlhe40/Bhlhe41*‐deficient AMs maintained their numbers and displayed normal expression level AM phenotype including CD11c, Siglec‐F, F4/80, and MHCII cell surface molecules. However, genome‐wide expression analysis of these *Bhlhe40/Bhlhe41*‐deficient AMs revealed the dysregulation of the AM expression program, as indicated by the upregulation of gene clusters that are expressed in other resident macrophages. In summary, the genome‐wide profiling of BACH2 and BHLHE40 binding in *ex vivo* AMs indicates that these factors can directly regulate AM identity.

### Gut and peritoneal macrophages

Gut and peritoneal cavity macrophages populate a specific part of the body enriched with microbiota‐ and diet‐derived factors including metabolites, micro‐ and macronutrients such as vitamin A/retinol. Retinoic acid (RA) is produced from retinol in subsequent enzymatic steps by gut epithelial and stromal cells [71], dendritic cells [72, 73], and macrophages [74] of the omentum in the gut mucosa [60]. RA‐dependent RARα activity induces the differentiation of the precursors of peritoneal macrophages by upregulating GATA6, a transcription factor associated with peritoneal‐macrophage‐specific genes including *Saa3*, *Lrg1*, *Arg1*, and *Prtn3* [2]. The crosstalk between the gut microbiota involves microbiota‐induced regulatory mechanisms in innate immune cell as well. For example, the basic leucine zipper protein NFIL3 regulates innate inflammatory responses against the enteric microbiota and is essential for maintaining gut homeostasis (Fig. 2C). Macrophage NFIL3 is identified as a regulatory transcription factor in macrophages *in vitro* and *in vivo* and controls *IL‐12b* expression at the promoter level induced by bacterial products and the enteric microbiota [75]. The *Il‐12b* promoter has a DNA‐binding element for NFIL3, and there is a basal and bacterial inflammation‐activated NFIL3 binding to this DNA element confirmed by chromatin immunoprecipitation (ChIP). In addition, colonic CD11b^+^ *lamina propria* mononuclear cells from Nfil3^−/−^ mice spontaneously express *Il‐12b* mRNA and lower expression of NFIL3 was observed in CD14^+^ *lamina propria* mononuclear cells from inflammatory bowel disease (IBD) patients compared with control subjects independently of the *Il‐12b* regulator [75] IL‐10 cytokine. These observations confirm that transcriptional repressors can play a critical role in maintaining physiological conditions.

The nuclear protein SP140 in colonic macrophages recognizes post‐translational modifications on histones and represses macrophage lineage‐inappropriate genes of gut macrophages [76]. SP140 preferentially occupies promoters of silenced, lineage‐inappropriate genes bearing the histone modification H3K27me3, such as the HOXA cluster in human macrophages, and ensures their repression. Depletion of SP140 in macrophages resulted in severe deficiencies in bacteria‐ and virus‐induced activation [76]. Moreover, this study also demonstrated that Crohn’s disease (CD) patients carrying *Sp140* SNPs displayed suppressed innate immune gene signatures in a mixed population of peripheral blood mononuclear cells compared to other CD patients. Hematopoietic stem cell‐specific knockdown of *Sp140* in mice resulted in exacerbated dextran sulfate sodium‐induced colitis, and low SP140 levels in human CD intestinal biopsies correlated with relatively lower intestinal inflammatory cytokine levels and improved the response to anti‐TNFα therapy. This study suggested that a loss of SP140 due to genetic variation contributes to a molecularly defined subset of CD characterized by ineffective mucosal innate immunity and gut homeostasis.

Evidence suggests that RA is a tissue‐derived signal, which instructs the localization and functional polarization of peritoneal macrophages by upregulating the expression level of the transcription factor GATA6 reversibly. GATA6 protein is a specific TF for peritoneal macrophages associated with the establishing of the tissue‐specific transcriptional and epigenetic landscape [3, 60]. Moreover, a previous report has shown that methyl transferase DNMT3A maintains a high expression of HDAC9 in a DNA methylation‐dependent manner in naïve peritoneal macrophages, and epigenetically prepares these cells to activate TBK1‐IRF3 signaling fully and produce interferon I after virus infection [77]. Nevertheless, macrophages in normal conditions are primed to respond rapidly and significantly to subsequent challenges, maintaining low levels of constitutive IFNβ and downstream Janus kinase (JAK)–STAT signaling. It is particularly noteworthy that the microbiota mimics the regulatory components of host protein networks. For example, the influenza A virus carries a sequence that resembles H3K4 and can block interactions with readers of H3K4me3, thereby suppressing the positive function of this epigenetic marker [78].

### Microglia

Microglia cells are a unique population of tissue‐resident macrophages that play essential roles in maintaining tissue homeostasis in the central nervous system (CNS). Microglial identity and function are mediated by the transcriptional factor Sall1 [3, 79], SMAD2/3, and IRF8 [80] and more recent studies also elucidated that negative transcriptional regulators play a role in microglial function in both health and disease. The Nr4a orphan nuclear receptors, Nr4a1 (Nur77), Nr4a2 (Nurr1), and Nr4a3 (Nor1), are early‐immediate response genes that can be induced by a variety of physiological stimuli such as inflammation during experimental autoimmune disease models. It was demonstrated that Nr4a1 directly suppresses the gene expression level of tyrosine hydroxylase (TH), the rate‐limiting enzyme for norepinephrine (NE) production in macrophages which enzyme protects mice from experimental autoimmune encephalomyelitis (EAE). Mechanistically, it was suggested that Nr4a1 could downregulate thyrosin‐hydroxylase (Th) gene transcription by recruiting the CoREST complex involving HDAC1 and HDAC2 enzymes in the Th promoter region [78] (Fig. 2D). This study also has shown that mice lacking Nr4a1 had a poor prognosis and had high concentrations of norepinephrine (NE), pro‐inflammatory IL‐6, and autoimmune effector T cells at the site of the affected tissue area of the CNS. ChIP analysis also showed increased abundance of acetylated histone H3 in the Th promoter following Nr4a1 knockdown and, to a higher extent, following CoREST knockdown.

## Conclusions and perspectives

Tissue macrophages are essential cellular components in maintaining tissue homeostasis and integrity through their professional phagocytic, antigen‐presenting, and self‐renewal ability ensuring the consistent protection and tissue regeneration of local tissue environment during infection and injury. Although the origin and the local tissue environment determine the epigenetic landscape and enhancer activity of macrophages, these innate immune cells can retain their polarization capacity depending on the actual extra‐ and intracellular signals. However, the molecular mechanisms regulating these processes are largely unknown. Inflammation is a typical physiologic process of innate and adaptive immunity during infection and injury to recruit effector immune cells including monocytes and tissue macrophages, which process requires transcriptional regulation in each step of the immune response. Intriguingly, the microbiota‐induced and maintained inflammatory response involves the precisely regulated activity of both transcriptional activators and repressors under homeostatic conditions in mucosal immunity, and unnecessary inflammation can be prevented. For example, the dysregulation of inflammatory and primary type I interferon signaling in pathogenic infections by respiratory viruses can be associated with macrophage activation syndrome and is typified by inflammatory cytokine‐driven alveolitis and thrombosis in the lungs [81]. Thus, determining the basal interaction level of lineage‐determining transcription factors and transcriptional repressors in different stages of macrophage development and polarization is critical to understand the molecular mechanisms of macrophage functions initiating inflammation and immune responses, resolution, and tissue repair, as well as for understanding the macrophage‐dependent pathology of chronic immune diseases and tumor development.

Some questions are raised regarding the transcriptional landscape of tissue macrophage biology. What kind of linage‐determining and signal‐dependent factor can interact with transcriptional repressors of different resident macrophages? What are the lineage‐specific target genes of transcriptional repressors in distinct tissue macrophages? How can macrophage‐dependent immune‐ and nonimmune disease states can affect the distribution of transcriptional repressors throughout the macrophage chromatin? A combination of new *in vivo* model systems, single cell‐based next‐generation gene sequencing (NGS), as well as novel innovative bioinformatic tools can enable us to uncover these questions and help to discover new therapeutic targets in macrophages.

## Conflict of interest

The authors declare no conflict of interest.

## Data Availability

The data that support the results of this study are available from the corresponding author upon reasonable request.

## References

[1] Glass CK and Natoli G (2016) Molecular control of activation and priming in macrophages. Nat Immunol 17, 26–33. 26681459 10.1038/ni.3306 PMC4795476

[2] Okabe Y and Medzhitov R (2016) Tissue biology perspective on macrophages. Nat Immunol 17, 9–17. 26681457 10.1038/ni.3320

[3] Lavin Y , Winter D , Blecher‐Gonen R , David E , Keren‐Shaul H , Merad M , Jung S and Amit I (2014) Tissue‐resident macrophage enhancer landscapes are shaped by the local microenvironment. Cell 159, 1312–1326. 25480296 10.1016/j.cell.2014.11.018 PMC4437213

[4] Nagy L , Szanto A , Szatmari I and Széles L (2012) Nuclear hormone receptors enable macrophages and dendritic cells to sense their lipid environment and shape their immune response. Physiol Rev 92, 739–789. 22535896 10.1152/physrev.00004.2011

[5] Murray PJ (2017) Macrophage polarization. Annu Rev Physiol 79, 541–566. 27813830 10.1146/annurev-physiol-022516-034339

[6] Hoeksema MA and Glass CK (2019) Nature and nurture of tissue‐specific macrophage phenotypes. Atherosclerosis 281, 159–167. 30343819 10.1016/j.atherosclerosis.2018.10.005 PMC6399046

[7] David Gosselin and Christopher Glass (2014) Epigenomics of macrophages. Immunol Rev 262, 96–112. 25319330 10.1111/imr.12213 PMC4203424

[8] Daniel B , Nagy G , Czimmerer Z , Horvath A , Hammers DW , Cuaranta‐Monroy I , Poliska S , Tzerpos P , Kolostyak Z *et al*. (2018) The nuclear receptor PPARγ Controls progressive macrophage polarization as a ligand‐insensitive epigenomic ratchet of transcriptional memory. Immunity 49, 615–626.e6. 30332629 10.1016/j.immuni.2018.09.005 PMC6197058

[9] Davies LC , Jenkins SJ , Allen JE and Taylor PR (2013) Tissue‐resident macrophages. Nat Immunol 14, 986–995. 24048120 10.1038/ni.2705 PMC4045180

[10] Haldar M , Kohyama M , So AYL , Kc W , Wu X , Briseño CG , Satpathy AT , Kretzer NM , Arase H , Rajasekaran NS *et al*. (2014) Heme‐mediated SPI‐C induction promotes monocyte differentiation into iron‐recycling macrophages. Cell 156, 1223–1234. 24630724 10.1016/j.cell.2014.01.069 PMC4010949

[11] Ogawa K , Sun J , Taketani S , Nakajima O , Nishitani C , Sassa S , Hayashi N , Yamamoto M , Shibahara S , Fujita H *et al*. (2001) Heme mediates derepression of Maf recognition element through direct binding to transcription repressor Bach1. EMBO J 20, 2835–2843. 11387216 10.1093/emboj/20.11.2835 PMC125477

[12] Bain CC and Mowat AM (2014) Macrophages in intestinal homeostasis and inflammation. Immunol Rev 260, 102–117. 24942685 10.1111/imr.12192 PMC4141699

[13] Neupane AS , Willson M , Chojnacki AK , Silva VE , Castanheira F , Morehouse C , Carestia A , Keller AE , Peiseler M , DiGiandomenico A *et al*. (2020) Patrolling alveolar macrophages conceal bacteria from the immune system to maintain homeostasis. Cell 183, 110–125.e11. 32888431 10.1016/j.cell.2020.08.020

[14] Hussell T and Bell TJ (2014) Alveolar macrophages: plasticity in a tissue‐specific context. Nat Rev Immunol 14, 81–93. 24445666 10.1038/nri3600

[15] Kang B , Alvarado LJ , Kim T , Lehmann ML , Cho H , He J , Li P , Kim BH , Larochelle A and Kelsall BL (2020) Commensal microbiota drive the functional diversification of colon macrophages. Mucosal Immunol 13, 216–229. 31772323 10.1038/s41385-019-0228-3 PMC7039809

[16] Charles JF and Aliprantis AO (2014) Osteoclasts: more than “bone eaters”. Trends Mol Med 20, 449–459. 25008556 10.1016/j.molmed.2014.06.001 PMC4119859

[17] Ajami B , Bennett JL , Krieger C , Tetzlaff W and Rossi FMV (2007) Local self‐renewal can sustain CNS microglia maintenance and function throughout adult life. Nat Neurosci 10, 1538–1543. 18026097 10.1038/nn2014

[18] Hashimoto D , Chow A , Noizat C , Teo P , Beasley MB , Leboeuf M , Becker CD , See P , Price J , Lucas D *et al*. (2013) Tissue‐resident macrophages self‐maintain locally throughout adult life with minimal contribution from circulating monocytes. Immunity 38, 792–804. 23601688 10.1016/j.immuni.2013.04.004 PMC3853406

[19] Schneider C , Nobs SP , Kurrer M , Rehrauer H , Thiele C and Kopf M (2014) Induction of the nuclear receptor PPAR‐γ by the cytokine GM‐CSF is critical for the differentiation of fetal monocytes into alveolar macrophages. Nat Immunol 15, 1026–1037. 25263125 10.1038/ni.3005

[20] Shaw TN , Houston SA , Wemyss K , Bridgeman HM , Barbera TA , Zangerle‐Murray T , Strangward P , Ridley AJL , Wang P , Tamoutounour S *et al*. (2018) Tissue‐resident macrophages in the intestine are long lived and defined by Tim‐4 and CD4 expression. J Exp Med 215, 1507–1518. 29789388 10.1084/jem.20180019 PMC5987925

[21] Wang J and Kubes P (2016) A reservoir of mature cavity macrophages that can rapidly invade visceral organs to affect tissue repair. Cell 165, 668–678. 27062926 10.1016/j.cell.2016.03.009

[22] Ginhoux F and Jung S (2014) Monocytes and macrophages: developmental pathways and tissue homeostasis. Nat Rev Immunol 14, 392–404. 24854589 10.1038/nri3671

[23] Zhang DE , Hetherington CJ , Chen HM and Tenen DG (1994) The macrophage transcription factor PU.1 directs tissue‐specific expression of the macrophage colony‐stimulating factor receptor. Mol Cell Biol 14, 373–381. 8264604 10.1128/mcb.14.1.373-381.1994 PMC358386

[24] Sarrazin S , Mossadegh‐Keller N , Fukao T , Aziz A , Mourcin F , Vanhille L , Kelly Modis L , Kastner P , Chan S , Duprez E *et al*. (2009) MafB restricts M‐CSF‐dependent myeloid commitment divisions of hematopoietic stem cells. Cell 138, 300–313. 19632180 10.1016/j.cell.2009.04.057

[25] Aziz A , Soucie E , Sarrazin S and Sieweke MH (2009) MafB/c‐Maf deficiency enables self‐renewal of differentiated functional macrophages. Science 326, 867–871. 19892988 10.1126/science.1176056

[26] Heath V , Suh HC , Holman M , Renn K , Gooya JM , Parkin S , Klarmann KD , Ortiz M , Johnson P and Keller J (2004) C/EBPα deficiency results in hyperproliferation of hematopoietic progenitor cells and disrupts macrophage development in vitro and in vivo. Blood 104, 1639–1647. 15073037 10.1182/blood-2003-11-3963

[27] Mancino A , Termanini A , Barozzi I , Ghisletti S , Ostuni R , Prosperini E , Ozato K and Natoli G (2015) A dual cis‐regulatory code links IRF8 to constitutive and inducible gene expression in macrophages. Genes Dev 29, 394–408. 25637355 10.1101/gad.257592.114 PMC4335295

[28] Gosselin D , Link VM , Romanoski CE , Fonseca GJ , Eichenfield DZ , Spann NJ , Stender JD , Chun HB , Garner H , Geissmann F *et al*. (2014) Environment drives selection and function of enhancers controlling tissue‐specific macrophage identities. Cell 159, 1327–1340. 25480297 10.1016/j.cell.2014.11.023 PMC4364385

[29] Baxter PS , Dando O , Emelianova K , He X , McKay S , Hardingham GE and Qiu J (2021) Microglial identity and inflammatory responses are controlled by the combined effects of neurons and astrocytes. Cell Rep 34, 108882. 33761343 10.1016/j.celrep.2021.108882 PMC7994374

[30] Juven‐Gershon T and Kadonaga JT (2010) Regulation of gene expression via the core promoter and the basal transcriptional machinery. Dev Biol 339, 225–229. 19682982 10.1016/j.ydbio.2009.08.009 PMC2830304

[31] Daniel B , Nagy G and Nagy L (2014) The intriguing complexities of mammalian gene regulation: How to link enhancers to regulated genes. Are we there yet? FEBS Lett 588, 2379–2391. 24945732 10.1016/j.febslet.2014.05.041

[32] Maston GA , Landt SG , Snyder M and Green MR (2012) Characterization of enhancer function from genome‐wide analyses. Annu Rev Genomics Hum Genet 13, 29–57. 22703170 10.1146/annurev-genom-090711-163723

[33] Whyte WA , Orlando DA , Hnisz D , Abraham BJ , Lin CY , Kagey MH , Rahl PB , Lee TI and Young RA (2013) Master transcription factors and mediator establish super‐enhancers at key cell identity genes. Cell 153, 307–319. 23582322 10.1016/j.cell.2013.03.035 PMC3653129

[34] Rao SSP , Huntley MH , Durand NC , Stamenova EK , Bochkov ID , Robinson JT , Sanborn AL , Machol I , Omer AD , Lander ES *et al*. (2014) A 3D map of the human genome at kilobase resolution reveals principles of chromatin looping. Cell 159, 1665–1680. 25497547 10.1016/j.cell.2014.11.021 PMC5635824

[35] Zhang DX and Glass CK (2013) Towards an understanding of cell‐specific functions of signal‐dependent transcription factors. J Mol Endocrinol 51, T37–T50. 24130129 10.1530/JME-13-0216 PMC4128342

[36] Yeh H and Ikezu T (2019) Transcriptional and epigenetic regulation of microglia in health and disease. Trends Mol Med 25, 96–111. 30578089 10.1016/j.molmed.2018.11.004 PMC6377292

[37] Treuter E , Fan R , Huang Z , Jakobsson T and Venteclef N (2017) Transcriptional repression in macrophages—basic mechanisms and alterations in metabolic inflammatory diseases. FEBS Lett 591, 2959–2977. 28902388 10.1002/1873-3468.12850

[38] Glass CK and Saijo K (2010) Nuclear receptor transrepression pathways that regulate inflammation in macrophages and T cells. Nat Rev Immunol 10, 365–376. 20414208 10.1038/nri2748

[39] King EM , Chivers JE , Rider CF , Minnich A , Giembycz MA and Newton R (2013) Glucocorticoid repression of inflammatory gene expression shows differential responsiveness by transactivation‐ and transrepression‐dependent mechanisms. PLoS ONE 8, e53936. 23349769 10.1371/journal.pone.0053936 PMC3545719

[40] Bougarne N , Paumelle R , Caron S , Hennuyer N , Mansouri R , Gervois P , Staels B , Haegeman G and De Bosscher K (2009) PPARα blocks glucocorticoid receptor α‐mediated transactivation but cooperates with the activated glucocorticoid receptor α for transrepression on NF‐κB. Proc Natl Acad Sci USA 106, 7397–7402. 19376972 10.1073/pnas.0806742106 PMC2678648

[41] Czimmerer Z , Daniel B , Horvath A , Rückerl D , Nagy G , Kiss M , Peloquin M , Budai MM , Cuaranta‐Monroy I , Simandi Z *et al*. (2018) The transcription factor STAT6 mediates direct repression of inflammatory enhancers and limits activation of alternatively polarized macrophages. Immunity 48, 75–90.e6. 29343442 10.1016/j.immuni.2017.12.010 PMC5772169

[42] Igarashi K , Kurosaki T and Roychoudhuri R (2017) BACH transcription factors in innate and adaptive immunity. Nat Rev Immunol 17, 437–450. 28461702 10.1038/nri.2017.26

[43] Barish GD , Yu RT , Karunasiri MS , Becerra D , Kim J , Tseng TW , Tai LJ , Leblanc M , Diehl C , Cerchietti L *et al*. (2012) The Bcl6‐SMRT/NCoR cistrome represses inflammation to attenuate atherosclerosis. Cell Metab 15, 554–562. 22465074 10.1016/j.cmet.2012.02.012 PMC3367511

[44] Sun J , Hoshino H , Takaku K , Nakajima O , Muto A , Suzuki H , Tashiro S , Takahashi S , Shibahara S , Alam J *et al*. (2002) Hemoprotein Bach1 regulates enhancer availability of heme oxygenase‐1 gene. EMBO J 21, 5216–5224. 12356737 10.1093/emboj/cdf516 PMC129038

[45] Patsalos A , Tzerpos P , Halasz L , Nagy G , Pap A , Giannakis N , Lyroni K , Koliaraki V , Pintye E , Dezso B *et al*. (2019) The BACH1–HMOX1 regulatory axis is indispensable for proper macrophage subtype specification and skeletal muscle regeneration. J Immunol 203, 1532–1547. 31405954 10.4049/jimmunol.1900553 PMC6736746

[46] Qiao Y , Kang K , Giannopoulou E , Fang C and Ivashkiv LB (2016) IFN‐γ Induces Histone 3 Lysine 27 Trimethylation in a Small Subset of Promoters to Stably Silence Gene Expression in Human Macrophages. Cell Rep 16, 3121–3129. 27653678 10.1016/j.celrep.2016.08.051 PMC5079287

[47] Medzhitov R and Horng T (2009) Transcriptional control of the inflammatory response. Nat Rev Immunol 9, 692–703. 19859064 10.1038/nri2634

[48] Li J (2000) Both corepressor proteins SMRT and N‐CoR exist in large protein complexes containing HDAC3. EMBO J 19, 4342–4350. 10944117 10.1093/emboj/19.16.4342 PMC302030

[49] Pascual G , Fong AL , Ogawa S , Gamliel A , Li AC , Perissi V , Rose DW , Willson TM , Rosenfeld MG and Glass CK (2005) A SUMOylation‐dependent pathway mediates transrepression of inflammatory response genes by PPAR‐γ. Nature 437, 759–763. 16127449 10.1038/nature03988 PMC1464798

[50] Blaschke F , Takata Y , Caglayan E , Collins A , Tontonoz P , Hsueh WA and Tangirala RK (2006) A nuclear receptor corepressor‐dependent pathway mediates suppression of cytokine‐induced C‐reactive protein gene expression by liver X receptor. Circ Res 99, 88–99. 10.1161/01.RES.0000252878.34269.06 17110595

[51] Li P , Spann NJ , Kaikkonen MU , Lu M , Oh DY , Fox JN , Bandyopadhyay G , Talukdar S , Xu J , Lagakos WS *et al*. (2013) NCoR repression of LXRs restricts macrophage biosynthesis of insulin‐sensitizing omega 3 fatty acids. Cell 155, 200–214. 24074869 10.1016/j.cell.2013.08.054 PMC4131699

[52] Eichenfield DZ , Troutman TD , Link VM , Lam MT , Cho H , Gosselin D , Spann NJ , Lesch HP , Tao J , Muto J *et al*. (2016) Tissue damage drives co‐localization of NF‐κB, Smad3, and Nrf2 to direct Rev‐erb sensitive wound repair in mouse macrophages. Elife 5, 1–30. 10.7554/eLife.13024 PMC4963201 27462873

[53] Lam MTY , Cho H , Lesch HP , Gosselin D , Heinz S , Tanaka‐Oishi Y , Benner C , Kaikkonen MU , Kim AS , Kosaka M *et al*. (2013) Rev‐Erbs repress macrophage gene expression by inhibiting enhancer‐directed transcription. Nature 498, 511–515. 23728303 10.1038/nature12209 PMC3839578

[54] Fan R , Toubal A , Goñi S , Drareni K , Huang Z , Alzaid F , Ballaire R , Ancel P , Liang N , Damdimopoulos A *et al*. (2016) Loss of the co‐repressor GPS2 sensitizes macrophage activation upon metabolic stress induced by obesity and type 2 diabetes. Nat Med 22, 780–791. 27270589 10.1038/nm.4114

[55] Huang Z , Liang N , Goñi S , Damdimopoulos A , Wang C , Ballaire R , Jager J , Niskanen H , Han H , Jakobsson T *et al*. (2021) The corepressors GPS2 and SMRT control enhancer and silencer remodeling via eRNA transcription during inflammatory activation of macrophages. Mol Cell 81, 953–968.e9. 33503407 10.1016/j.molcel.2020.12.040

[56] Czimmerer Z , Halasz L and Nagy L (2020) Unorthodox transcriptional mechanisms of lipid‐sensing nuclear receptors in macrophages: are we opening a new chapter? Front Endocrinol (Lausanne) 11, 1–13. 33362723 10.3389/fendo.2020.609099 PMC7758493

[57] Kiss M , Czimmerer Z , Nagy G , Bieniasz‐Krzywiec P , Ehling M , Pap A , Poliska S , Boto P , Tzerpos P , Horvath A *et al*. (2017) Retinoid X receptor suppresses a metastasis‐promoting transcriptional program in myeloid cells via a ligand‐insensitive mechanism. Proc Natl Acad Sci USA 114, 10725–10730. 28923935 10.1073/pnas.1700785114 PMC5635866

[58] Kohyama M , Ise W , Edelson BT , Wilker PR , Hildner K , Mejia C , Frazier WA , Murphy TL and Murphy KM (2009) Role for Spi‐C in the development of red pulp macrophages and splenic iron homeostasis. Nature 457, 318–321. 19037245 10.1038/nature07472 PMC2756102

[59] A‐Gonzalez N , Guillen JA , Gallardo G , Diaz M , de la Rosa JV , Hernandez IH , Casanova‐Acebes M , Lopez F , Tabraue C , Beceiro S *et al*. (2013) The nuclear receptor LXRα controls the functional specialization of splenic macrophages. Nat Immunol 14, 831–839. 23770640 10.1038/ni.2622 PMC3720686

[60] Okabe Y and Medzhitov R (2014) Tissue‐specific signals control reversible program of localization and functional polarization of macrophages. Cell 157, 832–844. 24792964 10.1016/j.cell.2014.04.016 PMC4137874

[61] Tacke R , Hilgendorf I , Garner H , Waterborg C , Park K , Nowyhed H , Hanna RN , Wu R , Swirski FK , Geissmann F and Hedrick CC (2015) The transcription factor NR4A1 is essential for the development of a novel macrophage subset in the thymus. Sci Rep 5, 10055. 26091486 10.1038/srep10055 PMC4473761

[62] Bories GFP , Yeudall S , Serbulea V , Fox TE , Isakson BE and Leitinger N (2020) Macrophage metabolic adaptation to heme detoxification involves CO‐dependent activation of the pentose phosphate pathway. Blood 136, 1535–1548. 32556090 10.1182/blood.2020004964 PMC7515686

[63] Zenke‐Kawasaki Y , Dohi Y , Katoh Y , Ikura T , Ikura M , Asahara T , Tokunaga F , Iwai K and Igarashi K (2007) Heme induces ubiquitination and degradation of the transcription factor Bach1. Mol Cell Biol 27, 6962–6971. 17682061 10.1128/MCB.02415-06 PMC2099246

[64] Itoh K , Chiba T , Takahashi S , Ishii T , Igarashi K , Katoh Y , Oyake T , Hayashi N , Satoh K , Hatayama I *et al*. (1997) An Nrf2/small Maf heterodimer mediates the induction of phase II detoxifying enzyme genes through antioxidant response elements. Biochem Biophys Res Commun 236, 313–322. 9240432 10.1006/bbrc.1997.6943

[65] Baird L and Yamamoto M (2020) The molecular mechanisms regulating the KEAP1‐NRF2 pathway. Mol Cell Biol 40, 1–23. 10.1128/MCB.00099-20 PMC7296212 32284348

[66] Tenhunen R , Marver HS and Schmid R (1968) The enzymatic conversion of heme to bilirubin by microsomal heme oxygenase. Proc Natl Acad Sci USA 61, 748–755. 4386763 10.1073/pnas.61.2.748 PMC225223

[67] Campbell NK , Fitzgerald HK and Dunne A (2021) Regulation of inflammation by the antioxidant haem oxygenase 1. Nat Rev Immunol 21, 411–425. 33514947 10.1038/s41577-020-00491-x

[68] Otterbein LE , Bach FH , Alam J , Soares M , Tao LuH , Wysk M , Davis RJ , Flavell RA and Choi AMK (2000) Carbon monoxide has anti‐inflammatory effects involving the mitogen‐activated protein kinase pathway. Nat Med 6, 422–428. 10742149 10.1038/74680

[69] Nakamura A , Ebina‐Shibuya R , Itoh‐Nakadai A , Muto A , Shima H , Saigusa D , Aoki J , Ebina M , Nukiwa T and Igarashi K (2013) Transcription repressor Bach2 is required for pulmonary surfactant homeostasis and alveolar macrophage function. J Exp Med 210, 2191–2204. 24127487 10.1084/jem.20130028 PMC3804940

[70] Rauschmeier R , Gustafsson C , Reinhardt A , A‐Gonzalez N , Tortola L , Cansever D , Subramanian S , Taneja R , Rossner MJ , Sieweke MH *et al*. (2019) Bhlhe40 and Bhlhe41 transcription factors regulate alveolar macrophage self‐renewal and identity. EMBO J 38, 1–20. 10.15252/embj.2018101233 PMC6769426 31414712

[71] Vicente‐Suarez I , Larange A , Reardon C , Matho M , Feau S , Chodaczek G , Park Y , Obata Y , Gold R , Wang‐Zhu Y *et al*. (2015) Unique lamina propria stromal cells imprint the functional phenotype of mucosal dendritic cells. Mucosal Immunol 8, 141–151. 24938743 10.1038/mi.2014.51 PMC4268120

[72] Gyöngyösi A , Szatmari I , Pap A , Dezso B , Pos Z , Széles L , Varga T and Nagy L (2013) RDH10, RALDH2, and CRABP2 are required components of PPAR γ‐directed ATRA synthesis and signaling in human dendritic cells. J Lipid Res 54, 2458–2474. 23833249 10.1194/jlr.M038984 PMC3735943

[73] Sun CM , Hall JA , Blank RB , Bouladoux N , Oukka M , Mora JR and Belkaid Y (2007) Small intestine lamina propria dendritic cells promote de novo generation of Foxp3 T reg cells via retinoic acid. J Exp Med 204, 1775–1785. 17620362 10.1084/jem.20070602 PMC2118682

[74] Sanders TJ , McCarthy NE , Giles EM , Davidson KLM , Haltalli MLR , Hazell S , Lindsay JO and Stagg AJ (2014) Increased production of retinoic acid by intestinal macrophages contributes to their inflammatory phenotype in patients with crohn’s disease. Gastroenterology 146, 1278–1288.e2. 24503130 10.1053/j.gastro.2014.01.057

[75] Smith AM , Qualls JE , O’Brien K , Balouzian L , Johnson PF , Schultz‐Cherry S , Smale ST and Murray PJ (2011) A distal enhancer in II12b is the target of transcriptional repression by the STAT3 pathway and requires the basic leucine zipper (B‐ZIP) protein NFIL3. J Biol Chem 286, 23582–23590. 21566115 10.1074/jbc.M111.249235 PMC3123121

[76] Mehta S , Cronkite DA , Basavappa M , Saunders TL , Adiliaghdam F , Amatullah H , Morrison SA , Pagan JD , Anthony RM , Tonnerre P *et al*. (2017) Maintenance of macrophage transcriptional programs and intestinal homeostasis by epigenetic reader SP140. Sci Immunol 2, eaag3160. 28783698 10.1126/sciimmunol.aag3160 PMC5549562

[77] Li X , Zhang Q , Ding Y , Liu Y , Zhao D , Zhao K , Shen Q , Liu X , Zhu X , Li N *et al*. (2016) Methyltransferase Dnmt3a upregulates HDAC9 to deacetylate the kinase TBK1 for activation of antiviral innate immunity. Nat Immunol 17, 806–815. 27240213 10.1038/ni.3464

[78] Chen S , Yang J , Wei Y and Wei X (2020) Epigenetic regulation of macrophages: from homeostasis maintenance to host defense. Cell Mol Immunol 17, 36–49. 31664225 10.1038/s41423-019-0315-0 PMC6952359

[79] Buttgereit A , Lelios I , Yu X , Vrohlings M , Krakoski NR , Gautier EL , Nishinakamura R , Becher B and Greter M (2016) Sall1 is a transcriptional regulator defining microglia identity and function. Nat Immunol 17, 1397–1406. 27776109 10.1038/ni.3585

[80] Minten C , Terry R , Deffrasnes C , King NJC and Campbell IL (2012) IFN Regulatory factor 8 is a key constitutive determinant of the morphological and molecular properties of microglia in the CNS. PLoS ONE 7, e49851. 23166780 10.1371/journal.pone.0049851 PMC3498170

[81] McGonagle D , Ramanan AV and Bridgewood C (2021) Immune cartography of macrophage activation syndrome in the COVID‐19 era. Nat Rev Rheumatol 17, 145–157. 33547426 10.1038/s41584-020-00571-1 PMC7863615

